# Supplementary material for: Influenza A Virus Infection Reactivates Human Endogenous Retroviruses Associated with Modulation of Antiviral Immunity
Source: Viruses. 2022 Jul 21;14(7):1591. doi: 10.3390/v14071591 (PMC9320126; doi:10.3390/v14071591)
Supplement: Supplementary file 1 [file viruses-14-01591-s001.zip › MotifAnalysis.zip!/MotifOutput_PR8M/homerResults/motif6.similar.html]

motif6

## Information for motif6

A
C
T
G
C
G
T
A
A
C
G
T
A
C
T
G
A
G
T
C
A
C
G
T
A
C
T
G
A
C
G
T
A
C
T
G
A
G
T
C
A
C
G
T
A
C
G
T
  
Reverse Opposite:  

C
G
T
A
C
G
T
A
A
C
T
G
A
G
T
C
C
G
T
A
A
G
T
C
C
G
T
A
A
C
T
G
A
G
T
C
C
G
T
A
A
C
G
T
A
G
T
C
  

|  |  |
| --- | --- |
| p-value: | 1e-17 |
| log p-value: | -3.977e+01 |
| Information Content per bp: | 1.530 |
| Number of Target Sequences with motif | 5.0 |
| Percentage of Target Sequences with motif | 21.74% |
| Number of Background Sequences with motif | 2.4 |
| Percentage of Background Sequences with motif | 0.01% |
| Average Position of motif in Targets | 151.6 +/- 5.5bp |
| Average Position of motif in Background | 150.7 +/- 20.2bp |
| Strand Bias (log2 ratio + to - strand density) | 10.0 |
| Multiplicity (# of sites on avg that occur together) | 1.00 |
| Motif File: | file (matrix) reverse opposite |

### Similar de novo motifs found

|  |  |  |  |  |  |  |  |
| --- | --- | --- | --- | --- | --- | --- | --- |
| Rank | Match Score | Redundant Motif | P-value | log P-value | % of Targets | % of Background | Motif file |
| 1 | 0.913 | A C T G C G T A A C G T A C T G A G T C A C G T A C T G A C G T A C T G A G T C | 1e-12 | -28.019961 | 21.74% | 0.05% | motif file (matrix) |
